# Performance Enhancement of MC-CDMA System through Novel Sensitive Bit Algorithm Aided Turbo Multi User Detection

**DOI:** 10.1371/journal.pone.0115710

**Published:** 2015-02-25

**Authors:** Rasadurai Kumaravel, Kumaratharan Narayanaswamy

**Affiliations:** Department of Information Technology, Sri Venkateswara College of Engineering, Pennalur—602117, Sriperumbudur, India; Beijing University, CHINA

## Abstract

Multi carrier code division multiple access (MC-CDMA) system is a promising multi carrier modulation (MCM) technique for high data rate wireless communication over frequency selective fading channels. MC-CDMA system is a combination of code division multiple access (CDMA) and orthogonal frequency division multiplexing (OFDM). The OFDM parts reduce multipath fading and inter symbol interference (ISI) and the CDMA part increases spectrum utilization. Advantages of this technique are its robustness in case of multipath propagation and improve security with the minimize ISI. Nevertheless, due to the loss of orthogonality at the receiver in a mobile environment, the multiple access interference (MAI) appears. The MAI is one of the factors that degrade the bit error rate (BER) performance of MC-CDMA system. The multiuser detection (MUD) and turbo coding are the two dominant techniques for enhancing the performance of the MC-CDMA systems in terms of BER as a solution of overcome to MAI effects. In this paper a low complexity iterative soft sensitive bits algorithm (SBA) aided logarithmic-*Maximum a-Posteriori* algorithm (Log MAP) based turbo MUD is proposed. Simulation results show that the proposed method provides better BER performance with low complexity decoding, by mitigating the detrimental effects of MAI.

## Introduction

MC-CDMA system is the strongest candidate for next generation wireless mobile communication systems due to its merits of high spectral efficiency and robustness against inter symbol interference (ISI) due to the presence of code division multiple access systems (CDMA) and orthogonal frequency division multiplexing (OFDM) systems [1]. The multi carrier modulation (MCM) techniques, the spreading is made in frequency domain and the time synchronization requirements are much lower than conventional direct sequence CDMA schemes. The conventional CDMA is not practical when the data transmission rates are very high [2–4]. To combat the difficulties faced by CDMA system and to improve the BER performance in multi user environment, MC-CDMA systems are used.

OFDM introduces multicarrier concept in CDMA to give rise to novel concept of MC-CDMA. MC-CDMA system has advantages of OFDM and CDMA. However poor orthogonality among the received spreading code sequence (SCS) results in introduction of the multiple access interference (MAI) [5–6]. MAI affects the BER performance of the MC-CDMA system. Many techniques have been proposed to improve the performance of MC-CDMA systems over fading channels, among them channel coding algorithms are the most admired technique. The maximum likelihood (ML) detector based optimal MUD is gives better BER performance in the MC-CDMA system. MUD techniques such as parallel and serial interference cancellation (PIC &SIC), minimum mean square error (MMSE) are considered. However the complexity of decoding is increased exponentially as the number of active users increases [7–8]. The advent of turbo codes has motivated a lot of research in MUD using iterative or turbo decoder techniques for MC-CDMA systems to reduce the complexity of decoding [9]. The powerful algorithm used in the turbo MUD is soft-input soft-output (SISO) algorithm. The SISO is implemented using one of two algorithms such as the soft output viterbi algorithm (SOVA) and the *Maximum a-Posteriori* (MAP) algorithm [10–13]. Both of these algorithms are related to the Viterbi algorithm, a common algorithm used to decode conventional convolutional codes [14]. The key distinction is that the Viterbi algorithm outputs hard bit decisions, the SOVA and MAP algorithms output soft decisions that gives in the form of a log-likelihood ratio (LLR) [10 & 20]. In this paper a low-complexity iterative MUD approach for MC-CDMA systems is proposed. This scheme effectively alleviates the harmful effects of MAI and confiscates the interference components from received signal by means of SOVA and Log MAP based turbo MUD Techniques. This scheme detects the signal based on *a posteriori probability* (APP) information and also performs iterative or turbo decoding for MAI cancellation. This turbo MUD significantly outperforms the conventional multiuser receiver for moderate and high signal-to-noise ratio.

The Log MAP algorithm is a SISO channel decoding technique, which takes soft input and produces the decoder output as soft decision. The receiver of MC-CDMA system could achieve good performance with ML and MAP based techniques, conversely its computational complexity still exist and it requires large number of iterations to achieve good system performance [15–19]. In order to reduce the computational complexity of Log MAP based MUD scheme, the MAP decoder is made to operate in the log domain [20]. To further reduce the complexity and to obtain enhanced system performance in terms of BER, this paper presents a novel low complexity method, a soft sensitive bits algorithm (SBA) aided Log MAP based turbo MUD. In this SBA aided Log MAP based Turbo MUD, the initial estimates of all the active user bits are obtained from a prior information of coded bits. By considering that a prior information detection estimated common or knowledge information error bits are refer as sensitive bits. The number of error bits in the initial estimated of a prior information detection coded bits vector is small than other detection methods. Therefore the number of common or knowledge information error bits (sensitive bits) is not expected large after the initial estimates of error bits. Let the total number of sensitive bits equal to *f*, the estimated sensitive bits of a posterior information detection is then used to identify sensitive bits. The sensitive bits are then defined as those bits corresponding to those identified coded bits vectors. Once the sensitive bits are identified, assume that the other remaining bits are correctly detected. Hence, the SBA estimates becomes well defined this will successfully reduce the complexity of the Log-MAP based turbo MUD. It is demonstrated that the SBA aided Log MAP based turbo MUD improves the BER performance with significantly reduce the computational complexity. Furthermore BER performance of proposed method outperforms the other conventional MAP and SOVA based turbo MUD schemes.

This paper is organized as follows. Section II describes the MC-CDMA system model. Section III presents the proposed Turbo MUD algorithms. The simulation results are discussed in section IV. The conclusion is described in section V.

## System Model

**Fig 1:**
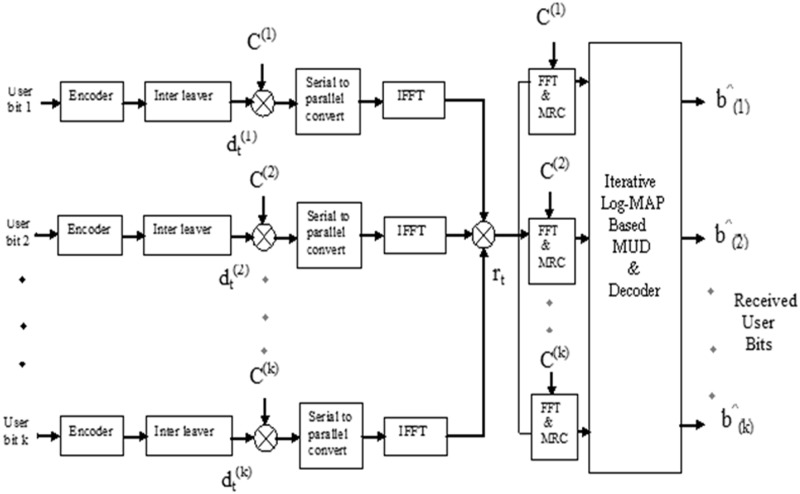
MC-CDMA system model.

A coded MC-CDMA system, with ‘*K*’ users assuming perfect frame synchronization is shown in Fig. 1. The information bits *b(k)* of the *k* ^th^ user is encoded using a convolutional encoder. The coded bit of the *k* ^th^ user at the *t* ^th^ time interval is then spread by a PN sequence and transmitted using MC-CDMA, where the total number of subcarriers is equal to the length of the signature sequence *L*. At the receiver, channel responses of the subcarriers are independent, the signal of each user is despread and maximum ratio combining (MRC) in the frequency domain is performed.

The received signal $\vec{r_{t}}$at the *t* ^th^ time interval is given by

$$\vec{r_{t}}=IFFT\{A_{t}G_{t}{\vec{d}}_{t}\}+n_{t}$$ (1)

where *A*_*t*_ is a *L* × *K* matrix with the *k* ^th^ column of *A*_*t*_ denoting a signal vector of *k* ^th^ user that includes the channel response and spreading codes in all carriers, ${\vec{d}}_{t}$ is the transmitted coded bits vector of *K* users and *n*_*t*_ is additive white Gaussian noise (AWGN). *G*_*t*_ denotes the average power of all users.

$$A_{t}=[\vec{s_{t,}^{1}}\vec{s_{t,}^{2}}......,\vec{s_{t}^{K}}]\vec{s_{t,}^{K}}={\left[\vec{s_{t,1}^{k}},\vec{s_{t,2}^{k}}......,{\vec{s_{t}^{k}}}_{,}{}_{L}\right]}^{T},\text{ k}=1,\ldots \ldots .,\text{K},\vec{s_{t,l}^{k}}=H_{t,l}^{k}.c_{l}^{\left(k\right)},\text{ l}=1,\ldots \ldots ...,\text{L}$$

Therefore,

$$G_{t}=\text{Diag}\left\{\sqrt{p_{t}^{1}},\sqrt{p_{t}^{2}},....,\sqrt{p_{t}^{k}}\right\}$$

where $H_{t,l}^{k}$ is the channel frequency response at the *l* ^th^ subcarrier of the *k* ^th^ user, and $p_{t}^{k}$is the received power of the *k* ^th^ user at the *t* ^th^ time interval. The matched filter (MF) output that includes the despreading and MRC operations, is written as

$$\begin{array}{c} {\vec{y}}_{t}=A_{t}^{H}.FFT\left\{IFFT\left\{A_{t}G_{t}\vec{d_{t}}\right\}+\vec{n_{t}}\right\}\\ =R_{t}G_{t}\vec{d_{t}}+v_{t} \end{array}$$ (2)

where *R*_*t*_ is the cross correlation matrix as$R_{t}=A_{t}^{H}A_{t}$, *ν*_*t*_ is complex Gaussian random process with zero mean and variance. The Equation (2) gives the received signal output${\vec{y}}_{t}$.

## Decoding Algorithms

The following section describes the two major classes of turbo decoding algorithms currently used to implement SISO decoders such as the soft output viterbi algorithm [11] (VA / SOVA) and the maximum a posteriori algorithm (MAP) also known as the BCJR algorithm, after Bahl, Cocke, Jelinek and Raviv. Both solutions are based on trellis coding method. The VA was designed to find the most probable *sequence* of states ***s*** with the given received symbols sequence ***y***

$$\hat{s}=\arg\{\underset{s}{\max}P[s|y]\}$$ (3)

The states found by the VA must form a connected path through the trellis. For this reason, the VA is the best method to minimize the frame error rate (FER) in communications systems. Conversely, the MAP is geared towards finding the most probable state *S*_*i*_ with the given received symbol *y*

$${\hat{s}}_{i}=\arg\{\underset{s}{\max}P[s_{i}|y]\}$$ (4)

The value of estimated sequence generated by the MAP need not be connected at all. The MAP is the best suited to minimize the bit error rate (BER). Turbo decoding works by independently estimating two individual processes. The two processes operate on the same data i.e., the second decoder uses an interleaved version of the original information. The decoding algorithm must take advantage of this fact by using one process output as a priori information for the other through soft-bit decisions. The end goal is to form LLRs which is used to estimate the bit sequence by performing hard decision on them.

$$\Lambda_{i}=\text{ln}(P[m_{i}=1|y]/P[m_{i}=0|y])$$ (5)

An expression for a binary phase shift keying (BPSK) modulated signal over a fading channel is considered. The binary sequence *X* = [100011…] to be sent over the fading channel. The channel impulse response of the received signal *y(t)*, is calculated as

y(t)=aX+n(t) (6)

where *a* represent the fading amplitude and *n(t)* is the AWGN detected at the receiver with variance *σ* ^2^ = *N*_*o*_ / 2*E*_*s*_. The LLR of SISO decoder output is expressed as sum of three entities

$$\Lambda_{i}=(2a_{i}/\sigma^{2})y_{i}{}^{\left(s\right)}+z_{i}+l_{i}$$ (7)

with *l*_*i*_ being the extrinsic information, *Z*_*i*_ is the information derived by the other decoder used as a priori information and the *y* term corresponding to the systematic observations. For the successful decoding of the information, the two SISO modules should exchange the extrinsic information exclusively.

### 1. Soft Output Viterbi Algorithm (SOVA)

The Viterbi algorithm is a decoding algorithm originally derived to ensure maximum likelihood detection of convolutional coded schemes. Trellis method represents the complete decoding process. The VA finds most likely path from the available received signal bits through the trellis method. Using Bayes’ theorem, becomes

$$\hat{s}=\arg\{\underset{s}{\max}P[y|s]P[s]/P[y]\}$$ (8)

Here the denominator in the above equation is same for all$\hat{s}$.

$$\hat{s}=\arg\{\underset{s}{\max}P[y|s]P[s]\}$$ (9)

To solve (9), the above equation is rewritten as

$$\hat{s}=\arg\{\underset{s}{\max}\sum_{i=0}^{L-1}\gamma (s_{i}\to s_{i+1})\}$$ (10)

$$\gamma (s_{i}\to s_{i+1})=\text{ln}P[y_{i}|s_{i}\to s_{i+1}]+\text{ln}P[s_{i+1}|s_{i}]$$ (11)

γ(*s*_*i* →_ *s*_*i*+1_) is the branch metric associated with the transition *s*_*i* →_ *s*_*i*+1_.The branch metric in terms of the transmitted symbols and messages that produce the state transitions is given as

$$\gamma (s_{i}\to s_{i+1})=\text{ln}P[y_{i}|x_{i}]+\text{ln}P[m_{i}]$$ (12)

where *m*_*i*_ and *x*_*i*_ are the message and output associated with the given state transition *s*_*i* →_ *s*_*i*+1_. γ(*s*_*i* →_ *s*_*i*+1_) is obtained from the a priori information *Z*_*i*_. For *m*_*i*_ = 1 *P*[*m*_*i*_] = {e^z^_i_ / 1 + e^z^_i_} and for *m*_*i*_ = 0 *P*[*m*_*i*_] = {1 / 1 + e^z^_i_}.

$$\text{ln}P[m_{i}]=z_{i}m_{i}-\text{ln}(1+e^{z_{i}})$$ (13)

In a flat fading environment, the branch metric is given as

$$\gamma (s_{i}\to s_{i+1})=z_{i}m_{i}-\{\text{ln}(1+e^{z_{i}})+0.5\text{ln}(\pi N_{0}/E_{s})\}-E_{s}/N0\sum_{q=0}^{n-1}[y_{i}{}^{\left(q\right)}-a_{i}{}^{\left(q\right)}(2x_{i}{}^{\left(q\right)}-1)]{}^{2}$$ (14)

When the signal to noise ratio of the noisy channel is small, the third term doesn’t factor heavily into the determination of *λ*. The first two factors rely on the extrinsic information produced by the other decoder that is used as a priori information in the current decoder. This will influence the branch metric heavily.

### 2. The Maximum A Posteriori Algorithm (MAP)

The MAP algorithm computes the APP of each state transition given the noisy observation at the receiver. There is a one to one correspondence between a state transition and its corresponding code symbol. The states connected by the MAP algorithm and state transition need not form a continuous path. The algorithm computes the APP of each possible state transition and chooses the one which is more likely (highest probability). In turbo decoding, the MAP finds the probabilities of individual message bit being either 1 or 0 with the given noisy observation *y*. It puts them into LLR form and this information is exchanged between the two decoders until the last iteration, at which point a hard decision is performed. The MAP algorithm starts by finding the probability of each valid state transition *P*[*s*_*i*_ → *s*_*i*+1_ ∣ *y*] given the noisy observation *y* and then the definition of conditional probability is used

$$P[s_{i}\to s_{i+1}|y]=P[s_{i}\to s_{i+1},y]/P[y]$$ (15)

where *P*[*s*_*i*_ → *s*_*i*+1_,*y*] is the joint probability of the state transition *s*_*i*_ → *s*_*i*+1_ and *y* the observation corrupted by noise. The numerator of the right term of (15) is partitioned into

$$P[s_{i}\to s_{i+1},y]=\alpha (s_{i})\gamma (s_{i}\to s_{i+1})\beta (s_{i+1})$$ (16)

where

$$\alpha (s_{i})=P[s_{i},(y_{0}......y_{i-1})]$$ (17)

$$\gamma (s_{i}\to s_{i+1})=P[s_{i+1},y_{i}|s_{i}]$$ (18)

$$\beta (s_{i+1})=P[(y_{i+1},......,y_{L-1})|s_{i+1}]$$ (19)

γ(*s*_*i*_ → *s*_*i*+1_) is the branch metric associated the transition *s*_*i*_ → *s*_*i*+1_.

$$\gamma (s_{i}\to s_{i+1})=P[s_{i+1}|s_{i}]P[y_{i}|s_{i}\to s_{i+1}]=P[m_{i}]P[y_{i}|x_{i}]$$ (20)

*P*[*y*_*i*_ ∣ *x*_*i*_] is a function of modulation and the channel model. According to Baye’s theorem.

$$P[y_{i}|x_{i}]=\frac{\mathrm{Pr}\{x_{i}|y_{i}\}pr\{x_{i}\}}{\mathrm{Pr}\{y_{i}\}}=C.\mathrm{Pr}\{x_{i}|y_{i}\}$$ (21)

where *C* is constant for a particular codeword and as a result is to be ignored for future calculations. The channel reliability factor$R_{i}{}^{\left(q\right)}$is

$$R_{i}{}^{\left(q\right)}=\text{ln}\left[\frac{pr\{x_{i}{}^{\left(q\right)}=1|y\}}{pr\{x_{i}{}^{\left(q\right)}=0|y\}}\right]R_{i}{}^{\left(q\right)}R_{i}{}^{\left(q\right)}$$ (22)

with *q* ϵ {0, …*n*-1} of a 1/*n* RSC encoder. Baye’s theorem dictates that the APP$\mathrm{Pr}\{x_{i}{}^{\left(q\right)}=b|y\},b\in \{0,1\}$, is expressed in terms of the APP,$\mathrm{Pr}\{y_{i}{}^{\left(q\right)}|x_{i}{}^{\left(q\right)}=b\}$. Since

$$\mathrm{Pr}\{y_{i}|x_{i}\}=\frac{1}{\sqrt{\pi \frac{N_{0}}{E_{s}}}}\exp\{-\frac{E_{s}}{N_{0}}[y_{i}{}^{\left(q\right)}-a_{i}{}^{\left(q\right)}(X_{i}{}^{\left(q\right)})]\}$$ (23)

Then the channel reliability factor is simplified as

$$R_{i}{}^{\left(q\right)}=4a_{i}{}^{\left(q\right)}\frac{E_{s}}{N0}y_{i}{}^{\left(q\right)}$$ (24)

The APPs$\mathrm{Pr}\{x_{i}{}^{\left(q\right)}=b|y_{i}{}^{\left(q\right)}$, *b* ∈ {0, 1} is given as

$$\mathrm{Pr}\{x_{i}{}^{\left(q\right)}|y_{i}{}^{\left(q\right)}\}=\frac{(eR_{i}{}^{\left(q\right)})x_{i}{}^{\left(q\right)}}{1+eR_{i}{}^{\left(q\right)}}$$ (25)

Consequently,

$$\gamma (s_{i}\to s_{i+1})=\frac{{\left(e^{z_{i}}\right)}^{m_{i}}}{1+e^{z_{i}}}\prod_{q=0}^{n-1}\frac{(eR_{i}{}^{\left(q\right)})x_{i}{}^{\left(q\right)}}{1+e^{R_{i}{}^{\left(q\right)}}}$$ (26)

The denominator of (26) remains constant for a given codeword. Then (26) is reduced to

$$\gamma (s_{i}\to s_{i+1})={\left(e^{z_{i}}\right)}^{m_{i}}\prod_{q=0}^{n-1}(eR_{i}{}^{\left(q\right)})x_{i}{}^{\left(q\right)}$$ (27)

The probability *α(s*_*i*_ *)* is found by forward recursion

$$\alpha (s_{i})=\sum_{s_{i-1}\in A}\alpha (s_{i-1})\gamma (s_{i-1}\to S_{i})$$ (28)

*A* is the set of all the states *s*_*i-1*_ connected to state *s*_*i*_. *β(s*_*i*_ *)* is found through backward recursion.

$$\beta (s_{i})=\sum_{s_{i-1}\in B}\beta (s_{i+1})\gamma (s_{i+1}\to S_{i})$$ (29)

where *B* is the set of states *s*_*i+1*_ connected to state *s*_*i*_. The log-likelihood Equation in (5) becomes

$$LLR=\Lambda_{i}=\text{ln}\left(\frac{\sum_{S_{1}}\alpha (s_{i})\gamma (s_{i}\to s_{i+1})\beta (s_{i+1})}{\sum_{S_{0}}\alpha (s_{i})\gamma (s_{i}\to s_{i+1})\beta (s_{i+1})}\right)$$ (30)

The MAP algorithm will calculate the APPs for each bit. Unfortunately the algorithm is computational intensive, and susceptible to round off errors. The errors can be alleviated by performing the MAP in the log domain. It becomes the Log MAP algorithm. Indeed, the LLRs consist of a sum of logarithms, are applied much earlier in the computations that changes multiplication into additions and divisions into subtractions. There are two algorithms that take advantage of this property of the logarithm, the Log MAP and the Max Log MAP. Consider the equation below

$$\text{ln}(e^{\delta 1}+....+e^{\delta n})\approx \underset{i\in \{1...n\}}{\max}\delta_{i}$$ (31)

The logarithm of the sum of exponentials is replaced by *n*-1 maximum operations on the arguments of the exponentials. This approximation is the characteristic of Max Log MAP algorithm, in which the branch metric becomes

$$\bar{\gamma }(s_{i}\to s_{i+1})=\text{ln}P[m_{i}^{}\}+\text{ln}P[y_{i}|x_{i}]=\text{ln}{\left(e^{z_{i}}\right)}^{m_{i}}+\overset{n-1}{\underset{q=0}{\sum }}\text{ln}{\left(e^{R_{i}{}^{\left(q\right)}}\right)}^{x_{i}{}^{\left(q\right)}}$$ (32)

The $\bar{\alpha }(s_{i})$ and $\bar{\beta }(s_{i})$ are expressed as

$$\bar{\alpha }(s_{i})=\text{ln}\;\alpha (s_{i})$$ (33)

$$\bar{\alpha }(s_{i})=\text{ln}\{\sum_{S_{i-1}\in A}\exp[\bar{\alpha }(s_{i-1})\bar{\gamma }(s_{i-1}\to s_{i})]\}-\underset{S_{i-1}\in A}{\max}[\bar{\alpha }(s_{i-1})]$$ (34)

$$\bar{\alpha }(s_{i})\approx \underset{S_{i-1}\in A}{\max*}[\bar{\alpha }(s_{i-1})+\bar{\gamma }(s_{i-1}\to s_{i})]$$ (35)

Here, the max* operator is simply equal to the maximum of the arguments. In a similar approach

$$\bar{\beta }(s_{i})=\text{ln}\beta (s_{i})$$ (36)

$$\bar{\beta }(s_{i})=\text{ln}\{\sum_{S_{i-1}\in A}\exp[\bar{\beta }(s_{i+1})\bar{\gamma }(s_{1}\to s_{i+1})]\}-\underset{S_{i-1}\in A}{\max}[\bar{\beta }(s_{i+1})]$$ (37)

$$\bar{\beta }(s_{i})\approx \underset{S_{i-1}\in A}{\max*}[\bar{\beta }(s_{i+1})+\bar{\gamma }(s_{i}\to s_{i+1})]$$ (38)

Once the $\bar{\alpha }(s_{i})$ and $\bar{\beta }(s_{i})$ can be found for all the states in the trellis, the LLR has the following form

$$\Lambda_{i}=\text{ln}\{\sum_{S_{1}}\exp[\bar{\alpha }(s_{i})\bar{\gamma }(s_{i}\to s_{i+1})\bar{\beta }(s_{i+1})]\}-\text{ln}\{\sum_{S_{0}}\exp[\bar{\alpha }(s_{i})\bar{\gamma }(s_{i}\to s_{i+1})\bar{\beta }(s_{i+1})]\}$$ (39)

$$\Lambda_{i}\approx \overset{\max}{s_{1}}*[\bar{\alpha }(s_{i})\bar{\gamma }(s_{i}\to s_{i+1})\bar{\beta }(s_{i+1})]-\overset{\max}{s_{0}}*[\bar{\alpha }(s_{i})\bar{\gamma }(s_{i}\to s_{i+1})\bar{\beta }(s_{i+1})]$$ (40)

Because of the approximate result of the Equation (31), the Max Log MAP is sub-optimal and yields inferior soft results compared to the Log MAP algorithm. The problem is to calculate exactly the logarithm of the sum of exponentials. Equation (31) is simplified by using the Jacobian logarithm [10].

$$\text{ln}(e^{\delta 1}+e^{\delta 2})=\max(\delta_{1}+\delta_{2})+f_{c}(|\delta_{1}-\delta_{2}|)$$ (41)

In (41), *f*_*c*_ is called the correction function, the difference in implementation between the Max Log MAP and the Log MAP. At each step made by the Max Log MAP, the correction function is applied, in effect of increasing the complexity. This is alleviated by storing values of *f*_*c*_ in a look up table. The table would only be a short, one dimensional because the computation is a function of the absolute value of the difference between *δ*_*1*_ and *δ*_*2*_. The algorithm for the Max Log MAP and the Log MAP is computed in three steps. First the forward recursion is found, to calculate the *α*_*s*_. Then the backward recursion is found, to calculate the *β*_*s*_. Finally, the results of forward and backward recursions are used to find the LLR.

### 3. Optimal MAP-based MUD Algorithm

As in the case of a conventional serial turbo code, the detector consists of two main parts, a MAP-based MUD structure and *K* parallel single-user MAP based decoders [13]. It is shown that iterations between the two parts separated by de-interleavers (*π* ^-1^) and interleavers (π) are performed. In this case, the two extrinsic informations $\lambda_{1e}^{k}$and $\lambda_{2e}^{k}$ of the *k* ^th^ user, from the MAP based MUD and single-user MAP based decoders, are exchanged respectively during the iterations. The MAP-based multiuser detector gives a posterior LLR of a transmitted “±1” for the code bit ${\vec{d}}_{t}$ of the *k* ^th^ user at the *t* ^th^ time interval. The LLR is given by

$$\Lambda_{1}(d_{t}^{\left(k\right)})≜\log\frac{P(d_{t}^{\left(k\right)}=+1|{\vec{y}}_{t})}{P(d_{t}^{\left(k\right)}=-1|{\vec{y}}_{t})}=\log\frac{p({\vec{y}}_{t}|d_{t}^{\left(k\right)}=+1)}{p({\vec{y}}_{t}|d_{t}^{\left(k\right)}=-1)}+\frac{P(d_{t}^{\left(k\right)}=+1|{\vec{y}}_{t})}{P(d_{t}^{\left(k\right)}=-1|{\vec{y}}_{t})},k=1,.....K$$ (42)

The first term in (42) is extrinsic information, which is derived from the MAP-based MUD and is denoted by$\lambda_{1e}^{k}$. So as to calculate the extrinsic information of the *k* ^th^ user$\lambda_{1e}^{k}$, the a priori information of all coded bits should be known which is denoted by$\lambda_{10}^{k}$ for 1^st^ iteration. The calculations of the extrinsic information in MAP-MUD are conducted in an iterative fashion with the given a priori information. The conditional likelihood probability distribution of ${\vec{y}}_{t}$with Gaussian probability density function is given by

$$p({\vec{y}}_{t}|d_{t}^{\left(k\right)}=d)=\frac{p({\vec{y}}_{t}|d_{t}^{\left(k\right)}=d)}{p(d_{t}^{\left(k\right)}=d)}=\underset{\vec{d_{t}:}d_{t}^{\left(K\right)}=d}{\sum^{\text{​}}}P_{r}\{{\vec{y}}_{t}|\vec{d_{t}\}}.\overset{i\neq k,i=k}{\underset{K}{\prod^{\text{​}}}}P_{r}\left\{d_{t}(i)\right\}$$ (43)

Since there is no priori information available, in the first iteration it is assumed that the coded bits are equally likely. In the following iterations, the a priori information of MAP based MUD is obtained from the extrinsic information delivered by the *k* ^th^ user’s channel decoder in a previous iteration as

$$\lambda_{10}^{k}=\lambda_{2e}^{k}$$ (44)

Finally, the channel decoder computes the a posteriori LLR of the information bits during the last iteration.

### 4. Proposed Soft Sensitive Bits Algorithm

The proposed sensitive bits algorithm greatly reduces the computational complexity of Log MAP based turbo MUD

and achieves good system performance. By obtaining initial estimates of all the user bits, some specific bits are identified and are referred as “sensitive bits”. These bits correspond to error bits that are most likely to be in error, assuming that all the other bits are correctly detected. By feeding the SBA estimates as the initial input to the turbo MUD, the initial estimates become well defined. This will effectively reduce the complexity of the Log MAP based turbo MUD.

Step1: Find the initial estimates from the received user symbol vector ${\vec{y}}_{t}^{\left(k\right)}$

Step2: Identify the Sensitive bits (Bits in error are said to be sensitive bits) *f* from the initial estimates of the received bit vector

Step3: Let *f* denotes the maximum number of sensitive bits in the proposed algorithm and assume the sensitive bits are at the position (*i*, *j*)^th^ bit of the bit vector *B*(*t*), where *i* = 1, … … . .*N* and *j* = *I*, *Q*

Step4: Update the initial estimates of the symbol vector by flipping or reversing the polarity of the sensitive bits. Keep the *f* as minimum that it should not exceed *f* < (2*k—f*).

Step5: Now use the Log MAP algorithm to obtain the user estimates among the 2 *f* possible symbol vectors which corresponds to the *f* sensitive bits.

Step6: After *I* ^th^ number of iterations the extrinsic information is updated to provide the final estimates.

It should be pointed out that the computational complexity of the sensitive bits aided Log MAP algorithm is mainly determined by the number of sensitive bits *f* [13].

### 5. Proposed Low complexity SBA aided Log MAP based turbo MUD

Log MAP based MUD is a SISO decoding technique. This decoding algorithm accepts soft inputs from the demodulator called a priori information and produces soft outputs called a posteriori information. The reliability of a decoded bit is represented by the APP. The original MAP algorithm [10, 11] is unsuitable for practical implementations because of the required multiplications and exponential operations. By formulating this algorithm in the logarithmic domain, the multiplications be converted into addition and exponentials are disappeared. However, become the soft combining operation, the estimation of user bits is much easier in Log MAP based turbo MUD. The use of SBA further reduces the complexity of the Log MAP based turbo MUD in MC-CDMA systems. It also improves the BER performance of the system with low complexity than the other conventional methods.

**Fig 2:**
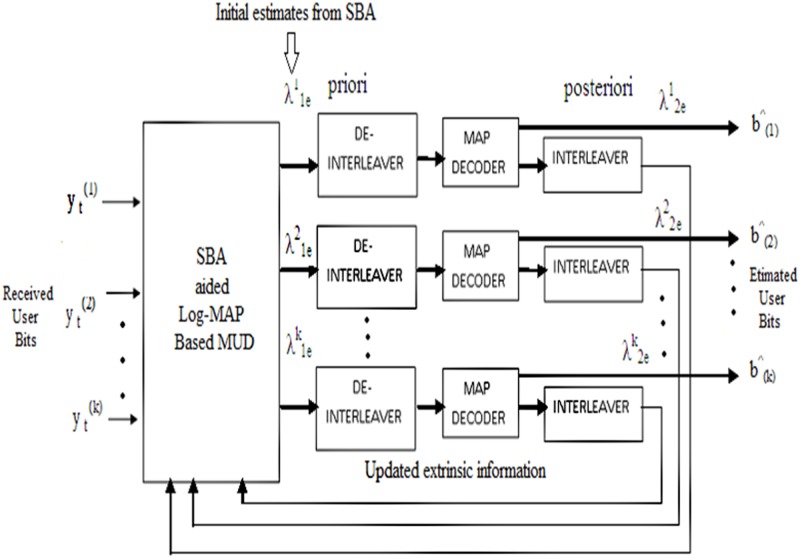
Proposed SBA aided Log MAP based turbo MUD.

Fig. 2 shows the proposed SBA aided Log MAP based turbo MUD. From the received user symbol vector, initial bits are found and then *f* sensitive bits are identified and updated by flipping and reversing the polarity of the error bits. These defined estimates are fed as an initial input to the Log MAP based turbo MUD [12, 13] as the first iteration intrinsic information. By turbo processing the estimates, sensitive bits are updated and better priori is achieved. The *k* ^th^ user channel decoder in a previous iteration from the received bits delivers the required extrinsic information${\vec{y}}_{t}^{\left(k\right)}$. The estimated priori $\lambda_{1e}^{k}$ information goes through iterative decoding process. The priori information is decoded by the MAP decoder and interleaved using a random interleaver, the estimated posteriori $\lambda_{2e}^{k}$ information is fed back to the SBA aided Log MAP section for further processing. The turbo MUD process is repeated for all K users. At the end of *I* ^th^ iterations the required extrinsic information LLR for *K* users will be found using SBA and Log MAP algorithms. The proposed sensitive bits algorithm [1, 14] greatly improves the performance of the MC-CDMA systems, by obtaining initial estimates of all the user coded bits, to identify some specific bits, which refer as “sensitive bits.” These bits correspond to error bits, assuming that all the other bits are correctly detected. In the next step the Log MAP algorithm is applied to correct these sensitive bits.

The initial estimates from the received symbol vector ${\vec{y}}_{t}^{\left(k\right)}$is denoted by$\hat{d}(t)={\left[\hat{d_{1}}(t),..............\hat{,d_{k}}(t)\right]}^{T}$. An initial estimated bit vector is given by *B*(*t*) = [*b*_1*I*_ (*t*), *b*_1*Q*_, …., *b*_*KI*_ (*t*), *b*_*KQ*_ (*t*)]^*T*^ the intrinsic information is updated by flipping or reversing the polarity of the sensitive bits *B*(*t*). Therefore the new vector *d* (*t*) is calculated, using the following metric

$$\Psi (\hat{d}(t))={\left(Y(t)-H(t)\tilde{d}(t)\right)}^{H}.(Y(t)-H(t)\tilde{d}(t))$$ (45)

The *f* reversed bits in the *f* selected $\Psi (\hat{d}(t))$are defined as “sensitive bits”. Then the residual 2*K*–*f* bits are fixed and the Log MAP algorithm is used to further detect the error bits. The final estimated bit vector consists of the combination of the 2*K*–*f* bits and the estimated *f* bits.

Thus, the a posteriori LLR of each coded bit of the *k* ^th^ user is given by [14]

$$\Lambda_{1}≜\log\frac{P(d_{t}^{\left(k\right)}=+1|\vec{y_{t})}}{P(d_{t}^{\left(k\right)}=-1|\vec{y_{t})}}\approx \lambda_{2e}^{k}+\lambda_{1e}^{k}$$ (46)

The extrinsic information delivered by the single user decoder is derived as

$$\lambda_{2e}^{k}=\Lambda_{2}-\lambda_{1e}^{k}$$ (47)

The required posteriori LLR needed is expressed as

$$\lambda_{2e}^{k}=\text{ln}(e^{a}+e^{b})\approx \max(a,b)+f_{c}-\lambda_{1e}^{k}$$ (48)

where the first term in Equation (48) is the LLR of the Log MAP algorithm and the Equation (48) is fed back to the Log MAP based turbo MUD to obtain the improved user estimates information. Specifically, the average bit error probability when varying the number of SBs *m* and number of iterations *n* against SNR. It is noted that, when number of SBs *m = K*, the proposed methods becomes equivalent to the optimal MAP based MUD with significant reduction in the system complexity.

Indeed, the proposed approach reduces the computational complexity of log MAP-based MUD from *O (K2* ^*K*^ *)* to *O ((K-f/2)2* ^*f*^ *)*, where *O* is computational complexity operation. Further, when *f* = 5 and n = 2 or 6 and, the performance of the proposed method is very close to that of the single user system, and the complexity is reduced from *O*(160) to *O*(28) [13]. It is also demonstrated that the proposed method is effective even when the number of SBs is much smaller than that of the number of users. The computational complexity of the Log MAP based MUD is greatly reduced from *O*(10240) to *O*(78) when *f* = 3 and *n* = 3 or 6 [13,14]. Hence, SBA aided Log MAP based turbo MUD is a novel method with low complexity approach that reduces the BER greatly by mitigating MAI. Subsequently the bandwidth of the channel can be utilized fairly to allocate more number of users.

## Simulation Results

The MC-CDMA system has been developed and simulated in Matlab version 7. The simulation parameters for the design and implementation of the system are given in Table 1. It is assumed that the receiver has perfect knowledge about the signal-to-noise ratio (SNR) and the noise variance. The encoder used is a turbo encoder with code rate = 1/n and constraint length K, which is combination of two recursive systematic convolution (RSC) coders which are joined by an interleaver and a feedback.

**Table 1:** Input Specification for MATLAB Simulation.

PARAMETER	SPECIFICATION
System	MC-CDMA
Users	10 users with 3 unknown users
Encoder	Turbo Coder
Decoder	SOVA, Log- MAP, SBA aided Log- MAP
Frame Size	1024
Code Rate	1/3
Constraint length	3
Iterations	2,4,6,8,10
Channel	AWGN, Rayleigh
Modulation	BPSK

For each simulation, a curve showing the BER versus the SNR per bit was computed. Also if the number of iterations is increased, the detector performs well that is, the BER performance of the system improved considerably. The simulated results of the proposed SBA aided Log MAP algorithm based turbo MUD for MC-CDMA system is demonstrated. Further the proposed system BER performances were analyzed and compared with SOVA based turbo MUD and Log MAP based turbo MUD respectively.

**Fig 3:**
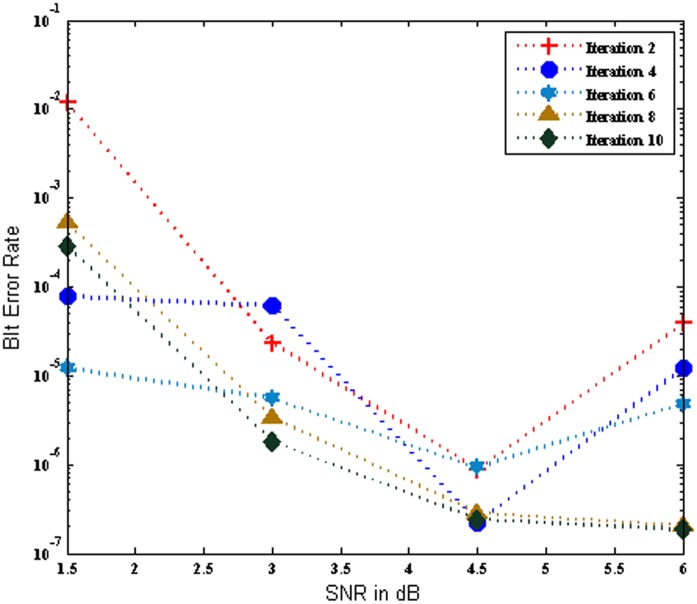
Performance of the MC-CDMA system using SOVA over AWGN channel.

Fig. 3 portrays the BER performance of the MC-CDMA system using SOVA algorithm based turbo MUD for various iterations over AWGN channel. An evaluation with 2, 4, 6, 8 and 10 iteration was performed and it is noticed that the performance of the system increases with increase in number of iterations. Further it is revealed that the SOVA based turbo MUD scheme achieved a maximum BER of 1.860E-07 at iteration 10 for SNR of 6dB. Also it is provided excellent BER performance of the system at low SNR value such as 1.5 dB to 4.5 dB.

**Fig 4:**
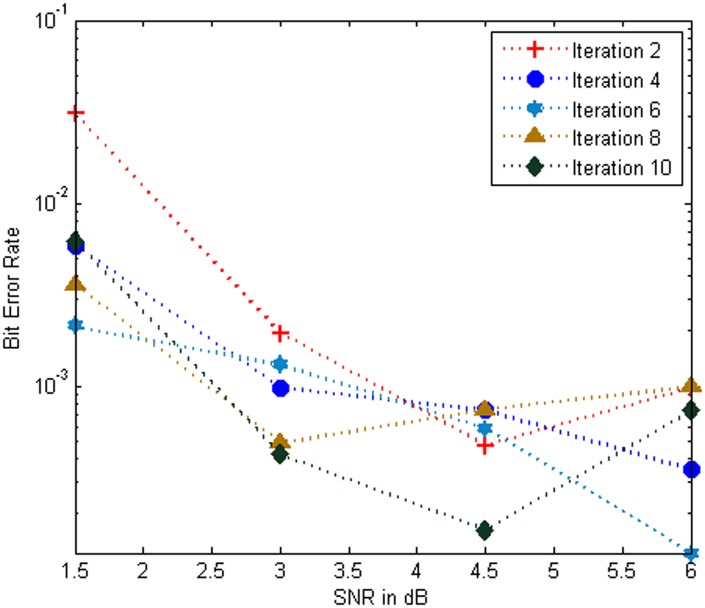
Performance of the MC-CDMA system using SOVA over Rayleigh Channel.

Fig. 4 renders the BER performance of the MC-CDMA system using SOVA algorithm based turbo MUD for various iterations over Rayleigh channel. In the Rayleigh channel the BER performance varies each time for same input parameters. It is evident that as the number of iterations increase with 2,4,6,8, and 10 iterations the BER performance of the system improved. It is notice that the analysis shows the Rayleigh channel provides much better BER for the low values of SNR of 1 dB to 4.5 dB. Further the SNR is increase, the BER performance of the system is slightly degraded for iteration 8 and iteration 10. Nevertheless, the Rayleigh channel introduces fading and more noise when compared to AWGN channel. Hence the BER performance of the system using SOVA based turbo MUD is degraded in Rayleigh channel.

**Fig 5:**
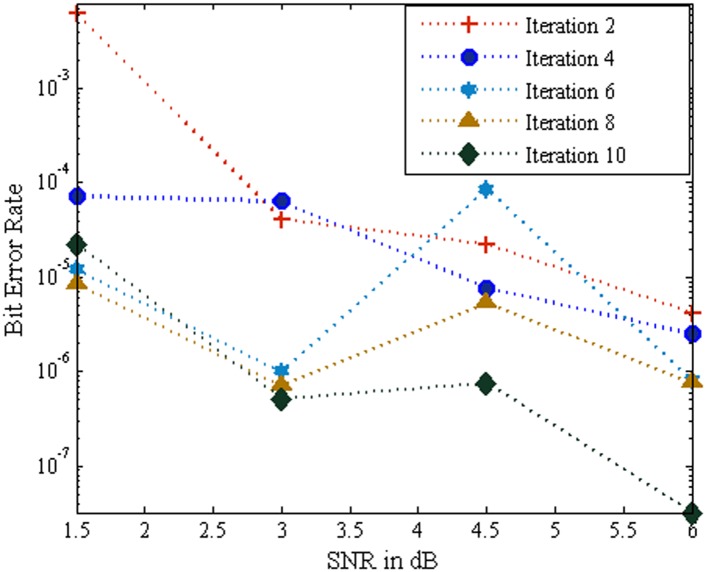
Performance of the MC-CDMA system using Log MAP over AWGN Channel.

Fig. 5 illustrates the BER performance of the MC-CDMA system using Log MAP algorithm based turbo MUD for various iterations over AWGN channel. It is observed from the plots that the number of iterations increases with 2,4,6,8 and 10 iteration the BER performance of the system improved progressively. It is clearly indicates that the Log MAP based turbo MUD outperform over SOVA based turbo MUD. Further it is revealed that the Log MAP based turbo MUD scheme yield a maximum BER of 3.21E-08 at iteration 10 for SNR 6 dB.

**Fig 6:**
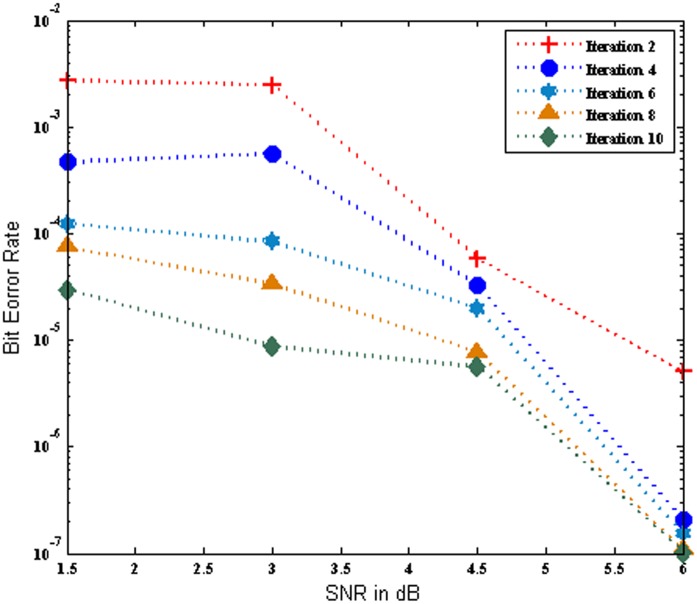
Performance of the MC-CDMA system using Log MAP over Rayleigh Channel.

Fig. 6 show that the BER performance of the MC-CDMA system using Log MAP algorithm based turbo MUD for various iterations over Rayleigh channel. It is noticed that as the number of iterations increases with 2,4,6,8 and 10 iteration the BER performance improved progressively. In the Fig. 5 and Fig. 6 shows that the performance of system using Log MAP over AWGN channel and Rayleigh channel respectively and it is provided the much better BER for the low values of SNR effectively from 1 dB to 6 dB. It is witnessed that the Log MAP based turbo MUD over AWGN channel outperforms Log MAP based turbo MUD over Rayleigh channel. Hence the BER performance of the system is slightly degraded in Rayleigh channel.

**Fig 7:**
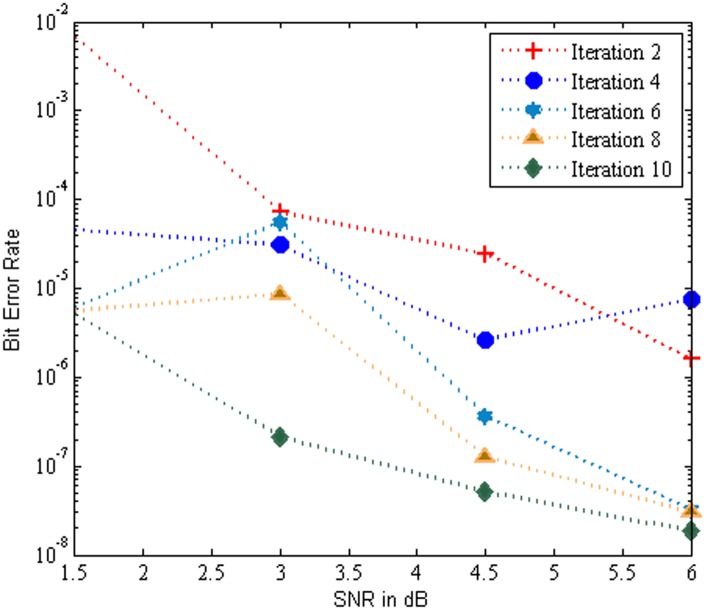
Performance of the MC-CDMA system using SBA aided Log MAP over AWGN Channel.

Fig. 7 illustrates the BER performance of the MC-CDMA system using SBA aided Log MAP algorithm based turbo MUD for various iterations over AWGN channel. An evaluation with 2,4,6,8 and 10 iterations was performed and it is revealed that the BER performance of the system increases with increase in number of iterations. The performance of the system yields a maximum BER value of 1.87E-08 at iteration 10 for SNR 6 dB. Also it is perform better BER performance at SNR value of 1.5 dB to 4.5 dB.

**Fig 8:**
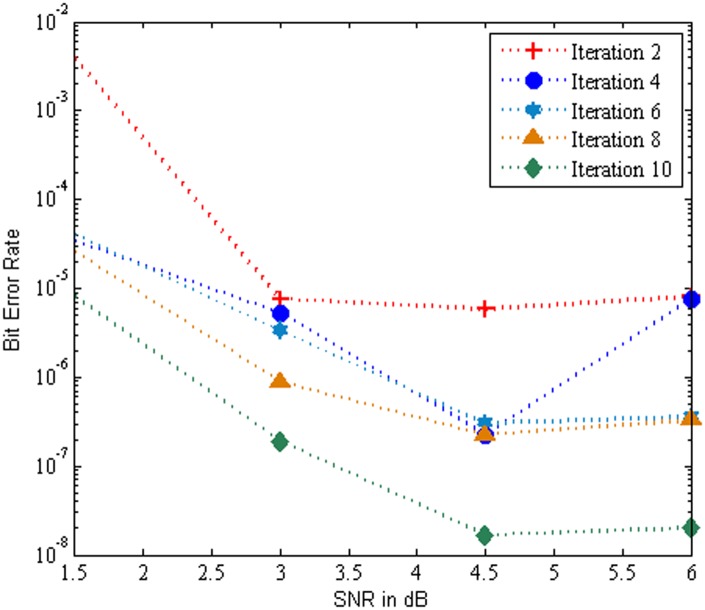
Performance of the MC-CDMA system using SBA aided Log MAP over Rayleigh Channel.

Fig. 8 show that the BER performance of the MC-CDMA system using SBA aided Log MAP algorithm based turbo MUD for various iterations over Rayleigh channel. An evaluation with 2,4,6,8 and 10 iterations was performed and it is clearly that the BER performance of the system increases with increase in number of iterations. Further the analysis shows that the Rayleigh channel provides much better BER performance at SNR of 6 dB. The performance of the system yields a maximum BER value of 1.684E-08 at iteration 10 for SNR 4.5 db.

**Fig 9:**
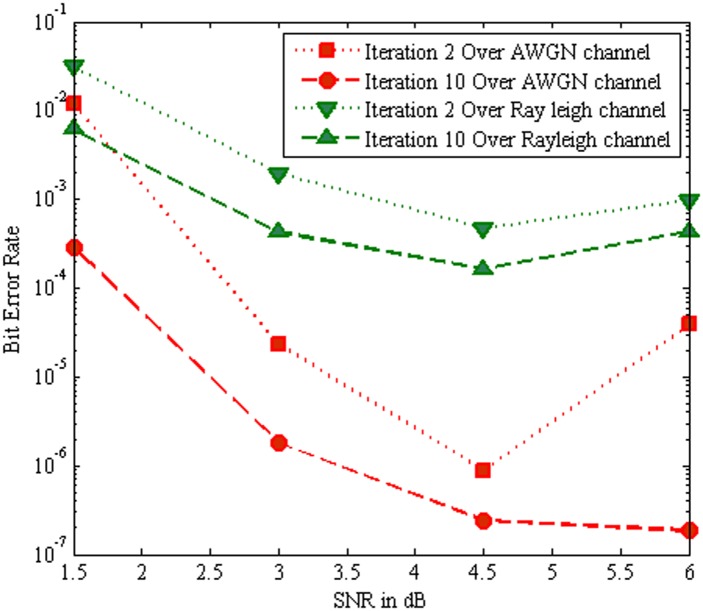
Performance comparison of SOVA algorithm with AWGN channel and Rayleigh Channel.

Fig. 9 show that the BER performance comparisons of the MC-CDMA system using SOVA algorithms for different iterations over AWGN channel and Rayleigh channel respectively. It is evident that as the number of iterations increases from iteration 2 to iteration 10 the BER performance of the system improved progressively. Nevertheless, the Rayleigh channel introduces fading and more noise when compared to AWGN channel. Hence the BER performance of the system is degraded in Rayleigh channel. Furthermore it is clear that the SOVA algorithm with AWGN channel gives better BER performance than SOVA algorithm with Rayleigh channel.

**Fig 10:**
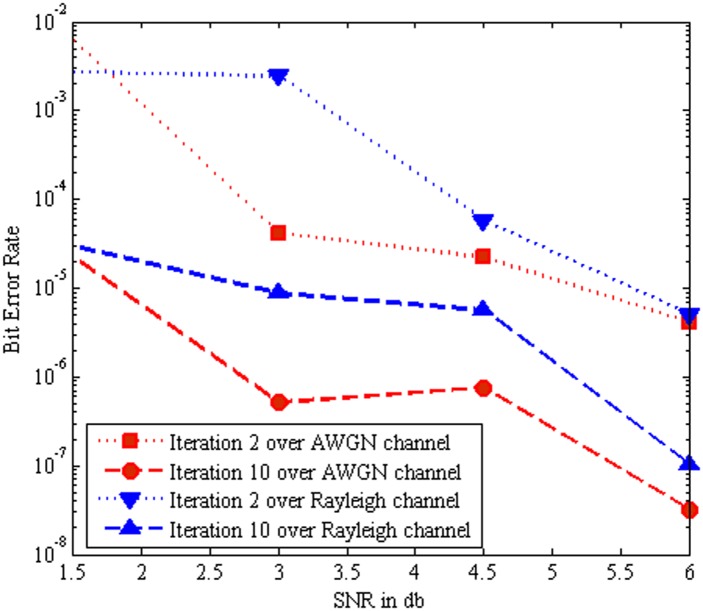
Performance comparison of Log MAP with AWGN channel and Rayleigh Channel.

Fig. 10 show that the BER performance comparison of the MC-CDMA system using Log MAP algorithm based turbo MUD over AWGN channel and Rayleigh channel respectively. It is evident that as the number of iterations increases from iteration 2 to iteration 10 the BER performance improved progressively. Nevertheless, the Rayleigh channel introduces fading and more noise when compared to AWGN channel. Hence the BER performance of the system is degraded in Rayleigh channel. Furthermore it is clear that the Log MAP algorithm with AWGN channel gives better BER performance than Log MAP algorithm with Rayleigh channel.

**Fig 11:**
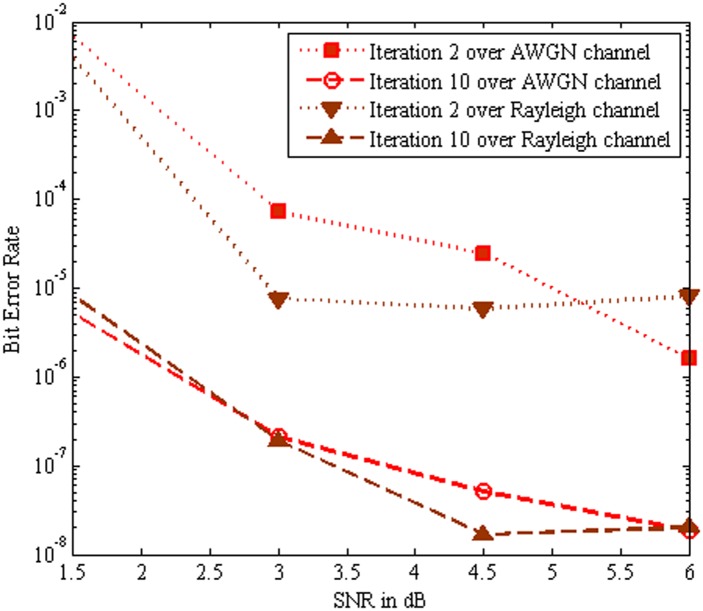
Performance comparison of SBA aided Log MAP over AWGN channel and Rayleigh Channel.

Fig. 11 show that the BER performance comparison of the MC-CDMA system using SBA aided Log MAP based turbo MUD over AWGN channel and Rayleigh channel respectively. Note that the simulation results of SBA aided Log MAP algorithm over Rayleigh channel at iteration 10 reflected the similar performance as predict by AWGN channel at iteration 10. It is portrayed that the SBA aided Log MAP algorithm is reduced computational complexity of the turbo MUD over Rayleigh channel.

**Fig 12:**
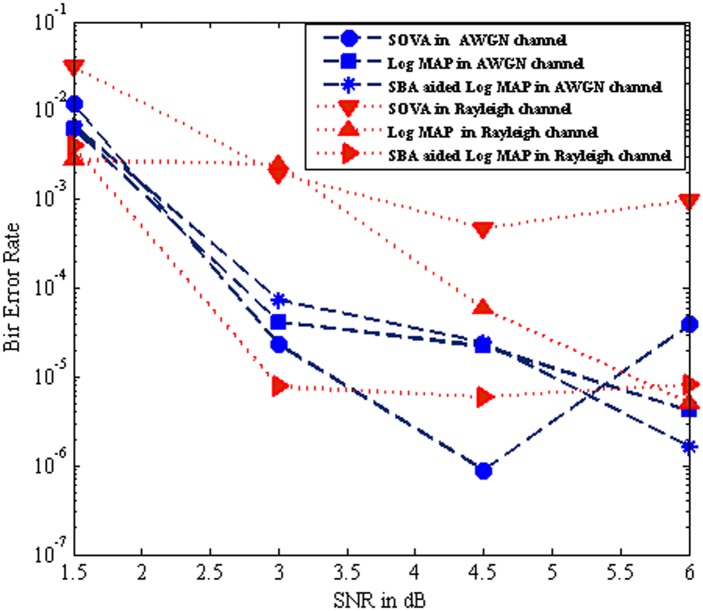
Performance comparison of SOVA, Log MAP and SBA aided Log MAP at Iteration 2.

Fig. 12 illustrates the BER performance comparison of the SOVA, Log MAP and SBA aided Log MAP algorithm for iteration 2 over AWGN channel and Rayleigh channel respectively. Results show that the SBA aided Log MAP based turbo MUD over Rayleigh channel achieved similar perform of the SOVA based AWGN channel at the low SNR low. Also the maximum BER performance is obtained in SBA aided Lop MAP at SNR of 4.5 dB. It is witnessed that the SBA aided Log MAP based turbo MUD performance much better than other turbo MUD.

**Fig 13:**
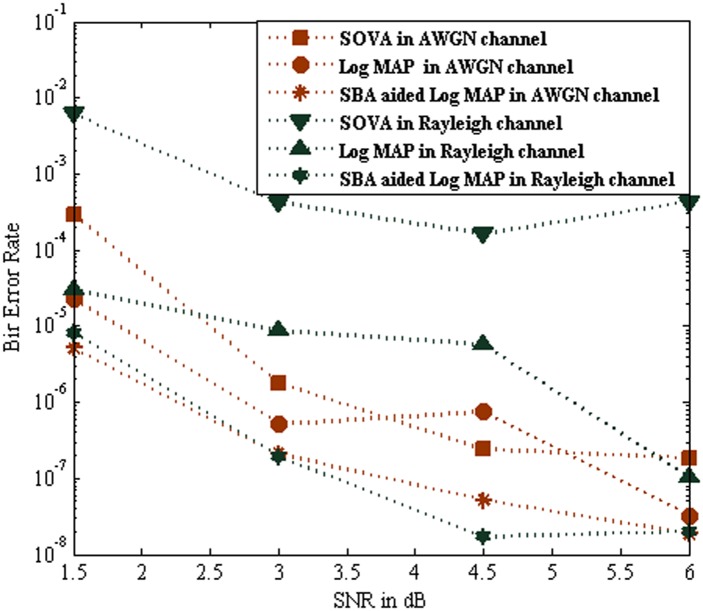
Performance comparison of SOVA, Log MAP and SBA aided Log MAP at Iteration 10.

Fig. 13 show that the BER performance comparison of the SOVA, Log MAP and SBA aided Log MAP algorithm for iteration 10 over AWGN channel and Rayleigh channel respectively. It is witnessed that the SBA aided Log MAP turbo MUD achieve excellent performance on both channels at iteration 10. Further it is observed the BER performance of SBA aided Log MAP turbo MUD over Rayleigh channel perform much better than Log MAP based turbo MUD. In Figs. 3, 4, 9, 12, and 13, it seems that the output of the SOVA algorithm is significantly more noisy than those from the Log MAP algorithm due to less robust modulations, coding schemes, channel reliability factor and also poor error correction performance [10–11]. It reveals that the SOVA algorithm curves can bring slight BER degradation in its error correction capacity, especially when high code rates and high SNR. This will be corrected further decoding iterations increase or by selecting the low code rates.

Table 2 and Table 3 shows that the BER performance of the MC CDMA system using SOVA for various iterations over AWGN channel and Rayleigh channel respectively. It clearly shows that the SOVA based turbo MUD over AWGN is better than SOVA based turbo MUD over Raleigh channel. The SOVA over AWGN channel yields a maximum BER of 1.860E-07 for the 10^th^ Iteration.

**Table 2:** BER Performance of the MC-CDMA system using SOVA based turbo MUD over AWGN channel.

Iteration / SNR in dB	SOVA based Turbo MUD over AWGN channel at Code Rate = 1/3			
	1.5	3	4.5	6
2	1.17E-02	2.30E-05	8.83E-07	3.866E-05
4	7.72E-05	6.14E-05	2.22E-07	1.202 E-05
6	1.21E-05	5.62E-06	9.33E-07	4.873 E-06
8	5.17E-04	3.35E-06	2.83E-07	2.060E-07
10	2.89E-04	1.81E-06	2.41E-07	1.860E-07

**Table 3:** BER Performance of the MC-CDMA system using SOVA based turbo MUD over Rayleigh channel.

Iteration / SNR in dB	SOVA based Turbo MUD over AWGN channel at Code Rate = 1/3			
	1.5	3	4.5	6
2	3.100E-02	1.9569E-03	4.7298E-04	9.7847E-04
4	5.870E-04	9.7847E-04	6.3386E-04	9.46E-04
6	2.120E-03	1.3044E-03	1.2231E-04	5.8708E-04
8	3.5877E-03	4.8924E-04	4.1173E-04	8.455E-04
10	6.1970E-03	4.24E-04	1.6308 E-04	4.34E-04

Table 4 and Table 5 shows that the BER performance of MC-CDMA system using Log MAP for various iterations over AWGN channel and Rayleigh channel respectively. The Log MAP over AWGN channel yields a maximum BER of 3.21E-08 for the 10^th^ Iteration. It clearly shows that the Log MAP based turbo MUD over AWGN is better than Log MAP based turbo MUD over Raleigh channel for low SNR. Furthermore, The SNR is 4.5 dB and above the BER performance of the system is nearly similar for both channels. Hence the Log MAP based Turbo MUD is suitable for practical condition.

**Table 4:** BER Performance of the MC-CDMA system using Log MAP based turbo MUD over AWGN channel.

Iteration / SNR in dB	Log MAP based Turbo MUD over AWGN channel at Code Rate = 1/3			
	1.5	3	4.5	6
2	6.20E-03	4.13E-05	2.22E-05	4.16E-06
4	7.22E-05	6.40E-05	7.73E-06	2.51E-06
6	1.20E-05	9.96E-07	8.47E-05	7.99E-07
8	8.52E-06	7.15E-07	5.40E-06	7.69E-07
10	2.25E-05	5.16E-07	7.50E-07	3.21E-08

**Table 5:** BER Performance of the MC-CDMA system using Log MAP based turbo MUD over Rayleigh channel.

Iteration / SNR in dB	Log MAP based Turbo MUD over Rayleigh channelat Code Rate = 1/3			
	1.5	3	4.5	6
2	2.72E-03	2.47E-03	5.798 E-05	5.07E-06
4	4.64E-04	5.63E-04	3.262 E-05	2.06E-07
6	1.24E-04	8.314 E-05	1.993 E-05	1.55E-07
8	7.481 E-05	3.339 E-05	7.749 E^—^05	1.08E-07
10	2.992 E-05	8.71 E^—^05	5.65E-06	1.03E-07

Table 6 and Table 7 shows that the BER performance of the MC-CDMA system using SBA aided Log MAP for various iterations over AWGN channel and Rayleigh channel respectively. The Log MAP over AWGN channel yields a maximum BER of 1.87E-08 for the 10^th^ Iteration. It clearly shows that the SBA aided Log MAP based turbo MUD over AWGN is better than Log MAP based turbo MUD over Raleigh channel for low SNR values. For SNR is above 4.5 the BER performance of the system is almost similar for both channels. Therefore the SBA aided Log MAP based turbo MUD is better than other turbo MUD methods.

**Table 6:** BER Performance of the MC-CDMA system using SBA aided Log MAP based turbo MUD over AWGN channel.

Iteration / SNR in dB	SBA aided Log MAP based Turbo MUD over AWGN channel at Code Rate = 1/3			
	1.5	3	4.5	6
2	6.70E-03	7.27E-05	2.46E-05	1.61E-06
4	4.543E-05	3.09E-05	2.59E-06	7.658E-06
6	6.093E-06	5.50E-05	3.67E-07	3.22E-08
8	5.563E-06	8.67E-06	1.27E-07	3.01E-08
10	5.136E-06	2.10E-07	5.14E-08	1.87E-08

**Table 7:** BER Performance of the MC-CDMA system using SBA aided Log MAP based turbo MUD over Rayleigh channel.

Iteration / SNR in dB	SBA aided Log MAP based Turbo MUD over Rayleigh channelat Code Rate = 1/3			
	1.5	3	4.5	6
2	3.9139E-03	7.6443E-06	5.8708E-03	8.1426E-06
4	3.496E-05	5.3226E-06	2.25E-07	7.6205E-06
6	4.0639E-05	3.3461E-06	3.0228E-07	3.5491E-07
8	2.5817E-05	8.9527E-07	2.2370E-07	3.2969E-07
10	8.1426E-06	1.92E-07	1.6843E-08	1.9985E-08

BER performance of the MC-CDMA using SOVA based turbo MUD, Log MAP based turbo MUD for Rayleigh channel and SBA aided Log MAP based turbo MUD Rayleigh channel are shown in Table 3, Table 5 and table 7 respectively. From the statistics values of BER performance of SBA aided Log MAP based turbo MUD Rayleigh channel for different iterations with its respective SNR values are much better over the Log MAP and SOVA based turbo MUD Rayleigh channel. The low complexity SBA aided Log MAP based Turbo MUD for Rayleigh channel yields a maximum BER of 1.6843E-08 for the 10^th^ Iteration of the code rate 1/3. From the comparison of tables 2, 3, 4, 5, 6 and 7 it conclusive that the lower iterations in all turbo MUD scheme the BER performance are similar to one other. At higher iterations and high SNR values the SBA aided Log MAP based turbo MUD scheme provides better BER performance over Log MAP based turbo MUD.

The SBA aided Log MAP turbo MUD scheme manages to pay the better BER of 10^–8^ and confirms nearly 0.5^–1^ BER improvements than Log MAP based MUD in average. Fig. 13 reveals BER performance of the MC-CDMA system as a function of SNR with *K* = 10 users. Specifically, the average bit error probability when varying the number of SBs *m* and the number of iterations *n* against SNR. It is noted that, when the number of SBs *m* = *K*, the proposed method becomes equivalent to the optimal MAP-based MUD. A close observation of Fig. 12 and Fig. 13 indicates that the performance of the proposed method can approach that of the optimal MAP-based MUD with significant reduction in system complexity.

## Conclusion

The next generation wireless communication system will be MAI liberated MC-CDMA systems with enhanced performance with improved channel capacity and high spectral efficiency in channel coding technique. Turbo MUD scheme is proposed for MC-CDMA system for improving the system performance in terms of BER. BER performance of the MC-CDMA using SOVA, Log MAP and SBA aided Lop MAP algorithm based Turbo MUD over AWGN channel and Rayleigh channel for different iterations are analyzed. It was observed that the BER performance of the system increases with the increase in number of iterations. An improved of computational complexity from *O(10240) to O(78)* was obtained when Log MAP was replaced with SBA aided Log MAP. This paper rendered as an effective low complexity scheme with a soft SBA aided Log MAP based turbo MUD for MC-CDMA systems that effectively reduces the MAI, computational complexity and enhanced BER performance. The result analysis reveals that the BER performance of proposed scheme approaches near optimum for the code rate of 1/3. The SBA aided Log MAP based turbo MUD over Rayleigh channel scheme affords a significant performance improvement with reduced complexity, hence it completely outperforms the other conventional optimal MAP based MUD schemes with low SNR utility and improved BER performance.
